# Host intestinal microbiota adaptive changes following *Paranosema locustae* infection and mechanism of chronic pathogenesis

**DOI:** 10.1093/jisesa/ieag027

**Published:** 2026-03-30

**Authors:** Huihui Zhang, Zhujun Cao, Xudong Zha, Weijing Wang, Roman Jashenko, Hongxia Hu, Rong Ji

**Affiliations:** Xinjiang Laboratory of Biology for the Protection and Regulation of Species in Special Environment, International Center for the Collaborative Management of Cross-border Pests in Central Asia, College of Life Sciences, Xinjiang Normal University, Urumqi, China; Tacheng, Research Field (Migratory Biology), Observation and Research Station of Xinjiang, Tacheng, China; Xinjiang Laboratory of Biology for the Protection and Regulation of Species in Special Environment, International Center for the Collaborative Management of Cross-border Pests in Central Asia, College of Life Sciences, Xinjiang Normal University, Urumqi, China; Tacheng, Research Field (Migratory Biology), Observation and Research Station of Xinjiang, Tacheng, China; Xinjiang Laboratory of Biology for the Protection and Regulation of Species in Special Environment, International Center for the Collaborative Management of Cross-border Pests in Central Asia, College of Life Sciences, Xinjiang Normal University, Urumqi, China; Tacheng, Research Field (Migratory Biology), Observation and Research Station of Xinjiang, Tacheng, China; Xinjiang Laboratory of Biology for the Protection and Regulation of Species in Special Environment, International Center for the Collaborative Management of Cross-border Pests in Central Asia, College of Life Sciences, Xinjiang Normal University, Urumqi, China; Tacheng, Research Field (Migratory Biology), Observation and Research Station of Xinjiang, Tacheng, China; Institute of Zoology, Ministry of Education and Science of Kazakhstan, Almaty, Kazakhstan; Xinjiang Laboratory of Biology for the Protection and Regulation of Species in Special Environment, International Center for the Collaborative Management of Cross-border Pests in Central Asia, College of Life Sciences, Xinjiang Normal University, Urumqi, China; Tacheng, Research Field (Migratory Biology), Observation and Research Station of Xinjiang, Tacheng, China; Xinjiang Laboratory of Biology for the Protection and Regulation of Species in Special Environment, International Center for the Collaborative Management of Cross-border Pests in Central Asia, College of Life Sciences, Xinjiang Normal University, Urumqi, China; Tacheng, Research Field (Migratory Biology), Observation and Research Station of Xinjiang, Tacheng, China

**Keywords:** *Paranosema locustae*, *Calliptamus italicus*, metagenome, intestinal microbe

## Abstract

*Paranosema locustae* infection reduces the abundance and diversity of the intestinal bacteria in locusts, although the microbial adaptive changes and the underlying mechanism of chronic pathogenesis remain unclear. In this study, the intestinal microbial changes in *Calliptamus italicus* (Linnaeus, 1758) (Orthoptera: Acrididae) were analyzed with metagenomic sequencing after *P. locustae* infection. Results showed that the diversity of intestinal microbial communities in *C. italicus* declined after *P. locustae* infection, while the abundance of infection-specific taxa in *C. italicus* in the experimental groups was significantly higher than those in the control groups, irrespective of sex (*P<*0.05). The populations of opportunistic pathogenic bacteria such as *Klebsiella aerogenes* and *Enterococcus faecalis* increased significantly (*P* < 0.05). Meanwhile, the abundances of probiotics such as *Pediococcus acidilactici* and *Enterobacter hormaechei* increased significantly (*P* <0.05), which could inhibit the pathogenicity of *P. locustae*. The results suggested that the interplay of changes in the species and quantities of probiotics and pathogenic bacteria in the intestine of *C. italicus* after *P. locustae* infection was an important factor contributing to the difficulty of *P. locustae* in quickly breaching the host defense system and to its chronic pathogenicity.

## Introduction

*Paranosema locustae* is a microbial species that obligately parasitizes Orthopterans, and by reducing the host’s feed intake, egg-laying capacity, and mobility, it can minimize pest damage. As a biological control agent, *P. locustae* is environmentally friendly and safe for humans and other nontarget organisms (Tan et al. 2015), and can be transmitted by the host in nature, achieving sustained population control of locusts. Therefore, *P. locustae* is widely used for the biological control of grassland locusts in high-incidence areas (Shi and Tan 2019, Huo et al. 2025).

Studies have shown that locusts infected with *P. locustae* do not die until 10 days after infection (Liu et al. 2023, Zhang et al. 2023). It is reported that *P. locustae* infection slows down the metabolism of *Locusta migratoria* (Linnaeus, 1758) (Orthoptera: Acrididae), as *P. locustae* inhibits the substances or pathways directly or indirectly involved in immune responses in vivo, which prolongs the incubation period of *P. locustae* in the host (Chen et al. 2020). The *P. locustae* infection can significantly alter the diversity, abundance, and composition of the intestinal microbial communities in *L. migratoria*, disrupting the structure of intestinal microbiota in the host (Tan et al. 2015), possibly since intestinal microbial communities play an important role in the immune defense of the host (Chen et al. 2020, Zhao et al. 2023). For example, the intestinal microbial communities of *Schistocerca gregaria* (Forsskaal, 1775) (Orthoptera: Acrididae) effectively inhibit *Metarhizium anisopliae* infection by secreting antifungal metabolites such as phenols, while its native intestinal bacteria can mediate colonization resistance and reduce susceptibility to pathogens by dually competing for ecological niches and ­regulating the host immune homeostasis (Dillon et al. 2005).

Existing studies mainly focus on the pathogenic mechanism of *P. locustae*, the immune responses of the host, and physiopathological changes during infection (Zeng et al. 2022, Kong et al. 2024, Guo et al. 2025). However, adaptive changes in the intestinal microbiomes of *Calliptamus italicus* in response to *P. locustae* infection and its mechanism of chronic pathogenesis remain unclear. In this study, the following scientific hypotheses were proposed: (i) *C. italicus* infection can induce adaptive changes in the composition of intestinal microbial communities in *C. italicus*. (ii) After *P. locustae* infection, the interactions between intestinal pathogenic bacteria and ­probiotics affect the chronic pathogenesis of *P. locustae*. To validate the above hypotheses, we infected *C. italicus* with *P. locustae* to study the host–pathogen interactions.

*C. italicus* is widely distributed worldwide and causes harm in European, the Mediterranean coast, North Africa, Central Asia, and West Asia abroad (Klein et al. 2021). In China, it is mainly distributed in Xinjiang, Inner Mongolia, Qinghai, and Gansu (Chen et al. 2024). It is a dominant, harmful species in the grasslands of Xinjiang, resulting in serious economic losses every year (Song et al. 2022), and is considered an ideal model species for studying insect–pathogen interactions.

## Materials and Methods

### Sample Collection and Feeding

*C. italicus* samples were collected from the Shirengou desert grassland (43°48′N, 87°50′E, 1,121 m above sea level) in Urumqi, Xinjiang, in August 2024 using the sweeping method. At the laboratory, the captured insects were placed in 15 × 15 × 40 cm insect cages, each with 5 samples. To ensure consistency, only healthy *C. italicus* individuals were selected for subsequent experiments (females: 0.75 ± 0.04 g in weight; males: 0.35 ± 0.03 g in weight [Supplementary Table S1]). They were fed twice a day (10:00 AM and 10:00 PM) with *Medicago falcata* L.(1753) planted in the experimental field. The host plants were rinsed with sterile water before feeding, and the insect cages were cleaned daily by removing feces and carcasses.

### Inoculation of *P. locustae*

*P. locustae* was diluted to 1 × 10^8^ spores/ml with a blood counting chamber under an optical microscope, and preserved at 4 °C for later use. The test insects were starved for 12 h before inoculation, and 5 µl of the well-mixed *P. locustae* diluent was extracted with a pipette and slowly applied to the mouthparts of each healthy nymph locusts for ingestion. Uninfected, healthy locusts served as controls. The experimental and control groups were kept separately under consistent conditions (temperature: 28 °C; relative humidity: 50%; photoperiod: 14 L:10 D). The stock solution of *P. locustae* was provided by the Key Open Laboratory of the Ministry of Agriculture and Rural Affairs for Biological Control at China Agricultural University.

### Sample Collection

According to the research results of Zhang et al. (2023), the test insects in this study were starved for 12 d after infection with *P. locustae*, followed by sampling on the 13th day. The polypide was first soaked in 75% alcohol for 2 min to disinfect it, and was then rinsed with sterile water. It was placed on an ultraclean workbench, fixed on a dissecting tray and quickly dissected by removing its feet and wings with sterilized dissecting scissors and cutting open its abdomen along one side of the anus. The intestinal tract was collected and placed in a 2 ml cryovial for liquid nitrogen quick freezing. After that, the cryovial was immediately transferred to a −80 °C freezer for later use. Every 4 locusts served as a biological replicate, and 3 biological replicates were prepared for females and males, respectively.

### Infection Identification and Sequencing

Intestinal DNA samples were extracted following the instructions of the HiPure Stool/Soil DNA Mini Kit (Tiangen, China). Using DNA as the template, infection was identified with a TaqMan real-time quantitative PCR (qPCR). The primers (forward: 5ʹ-CCGGAGGATCAAAGATGATTAGA-3′ʹ; reverse: 5ʹ-CCGTCGGCATCGTTTACTG-3′ʹ) and the Taq Man probe (5ʹ-ACCGTCGTAGTTCCG-3′ʹ) were designed in Primer Premier 5.0 with reference to the 16S ribosomal RNA gene sequence of *P. locustae* (Gen Bank ID: AY305324). The 5′-terminal fluorescent group of the Taq Man probe was 6-carboxyfluorescein (FAM), and the 3′-terminal fluorescent quenching group was 6-carboxytetramethylrhodamine (TAMRA). Both the primers and the probe were synthesized by Sangon Biotech Co., Ltd (Shanghai). The Taq Man qPCR reaction system was as follows: 10 μl Premix Ex Taq (Takara, Japan), 0.4 μl each of upstream and downstream primers (10 μM), 0.05 μl of the probe (10 μM), 100 ng of the DNA template, and RNase-free ddH_2_0 added to 20 μl. Amplification followed the following sequence: 95 °C for 3 min, 95 °C for 10 s, and 60 °C for 1 min, for a total of 40 cycles. Each PCR procedure included a positive control and a negative control for the DNA of *P. locustae*. Samples were sent to Shanghai Honsun Biological Technology Co., Ltd for metagenomic sequencing on the Illumina PE150 platform.

### Bioinformatics Analysis

The raw sequencing data were processed with Readfq (https://github.com/cjfields/readfq) to remove low-quality bases (threshold < 20, length < 50 bp) and reads containing ambiguous bases. MEGAHIT was used for metagenomic assembly to obtain longer overlapping sequences (Contigs). Prodigal was adopted to identify open reading frames (ORFs), predict coding sequences (CDS) regions, and acquire the gene sequences of each sample. The initial gene catalog was constructed with MMseqs2 redundancy filtering (identity = 95%; coverage = 90%), and the clean data were aligned to the initial gene set in Bowtie2 (http://bowtie-bio.sourceforge.net/bowtie2/index.shtml), to produce the final Unigenes. DIAMOND (https://github.com/bbuchfink/diamond/) was used to compare the generated Unigenes with the NCBI NR database (https://www.ncbi.nlm.nih.gov/) to obtain species annotation information, and with the KEGG database (http://www.kegg.jp/kegg/) providing functional annotation information.

### Data Analysis

Alpha diversity analysis was performed by using species-level annotation information statistics. Diversity indices included Chao1, Shannon, and Simpson, and intraspecific differences were analyzed with the Wilcoxon test. For Beta diversity indicators, principal coordinates analysis (PCoA) and analysis of similarity were employed to determine intergroup differences in the structure of intestinal microbial communities. UpSet analysis (threshold > 0.1) was conducted to test the populations of common and specific microbes in the intestinal microbial communities of insects in each group. Linear discriminant ­analysis effect size (LEfSe) analysis was performed to identify the taxa with the greatest difference in abundance between the 2 groups.

## Results

### Metagenomic and Diversity Analysis of Intestinal Microbial Communities

According to the detection results of the Taq Man qPCR (Table 1), the samples in the male and female infected groups were positive, while no *P. locustae* was detected in either control group. This indicated that the test insects were successfully infected with *P. locustae*, and a *C. italicus–P. locustae* infection model was built.

**Table 1.** Detection results of Taq Man qPCR after the infection of *C. italicus* with *P. locustae*

Groups	Female	Male	±
	Mean ± S.D.	Mean ± S.D.	
**Infection group**	22.55 ± 0.86	21.60 ± 0.56	+
**Control group**	—	—	−
**Positive control**	16.41 ± 0.12		+
**Negative control**	—		−

“+” represents a positive result, meaning that *P. locustae* was detected in the DNA sample of *C. italicus*; “−” represents a negative result, meaning that no *P. locustae* was detected in the DNA sample of *C. italicus*.

Clean Data from the intestinal samples of male and female *C. italicus* before and after *P. locustae* infection were obtained by sequencing on the Illumina HiSeq platform (Supplementary Table S2). All Clean Data were assembled with Megahit, yielding 1,369,105, 1,337,039, 1,114,009, and 1,307,224 contigs, respectively (Supplementary Table S3).

The sequencing results of the 4 groups of samples, ie the female *C. italicus* infected group (FI), the female *C. italicus* control group (FC), the male *C. italicus* infected group (MI), and the male *C. italicus* control group (MC), were annotated to bacterial, fungal, viral, and archaeal communities, as shown in Table 2. Bacterial communities had the highest relative abundance, with values of 92.41%, 81.32%, 69.33%, and 70.75% in FI, FC, MI, and MC, respectively. Fungi ranked second, and the abundance of fungi in males was higher than that of females. The relative abundances of viruses in the male and female infected groups were lower than those in the control groups, respectively. The Archaea had the lowest abundances, with a mean relative abundance of <0.5%.

**Table 2.** Changes in the composition of intestinal microbial communities before and after infection of *C. italicus* with *P. locustae*

Kingdom	Relative abundance			
	FI (%)	FC (%)	MI (%)	MC (%)
**Bacteria**	92.41	81.32	69.33	70.75
**Eukaryota**	4.55	11.53	24.36	19.45
**Virus**	2.99	7.05	6.09	9.58
**Archaea**	0.05	0.10	0.22	0.22

**Fig. 1.**
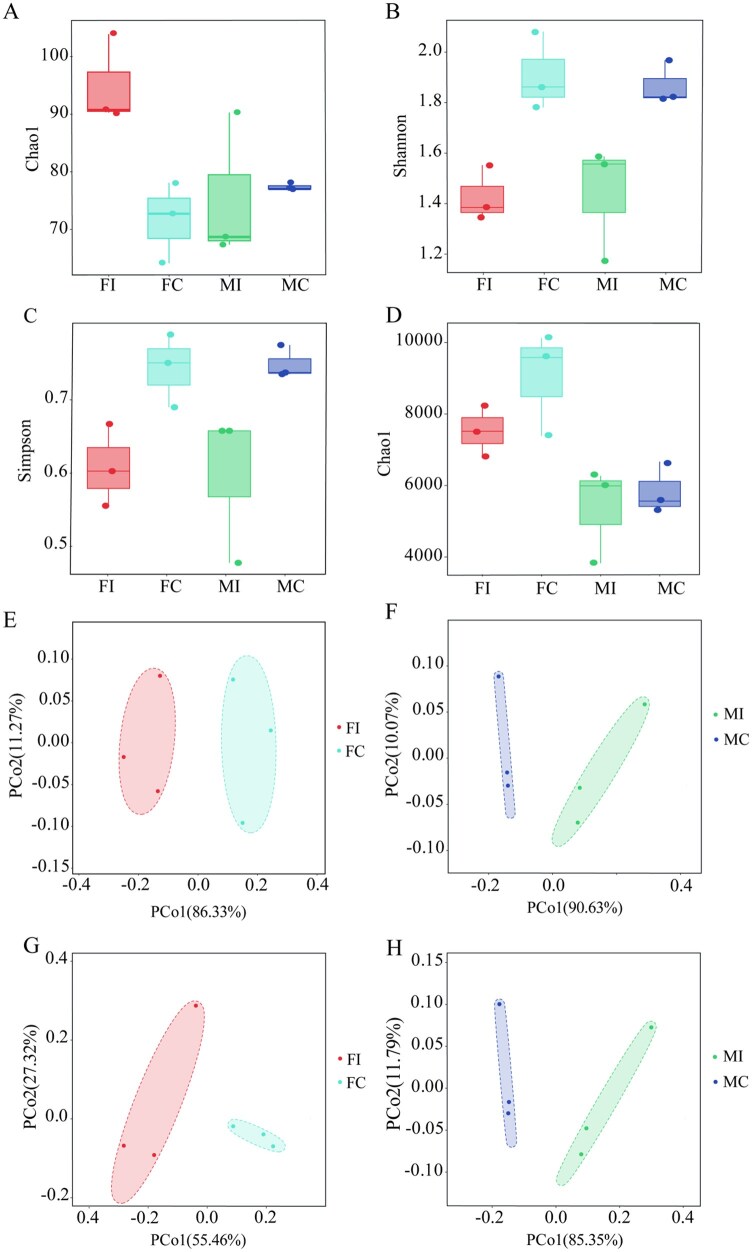
Diversity analysis of intestinal microbial communities after the infection of *C. italicus* with *P. locustae*. Diversity analysis of intestinal microbial communities at the phylum level in FI, FC, MI, and MC. (A–D) Alpha diversity analysis [Chao1 (A), Shannon (B), and Simpson (C) indices at the phylum level and Chao1 (D) index at the species level]. (E–H) PCoA, at the phylum level (E, F) and the species level (G, H), showing intergroup differences.

According to the Alpha diversity analysis of the intestinal microbial communities before and after the infection with *P. locustae*, FI had the highest richness of intestinal microbial communities at the phylum level (Fig. 1A). At the species level, the richness of intestinal microbes in females was higher than that in males (Fig. 1D), but the differences were nonsignificant (Wilcoxon test, *P* >0.05). The evenness and diversity of intestinal microbial communities in the infected groups (FI, MI) were lower than those in the control groups (FC, MC) (Fig. 1B and C), respectively. Data suggests that *P. locustae* infection reduced the diversity of intestinal microbial communities in the host.

The PCoA based on the Bray-Curtis distance (Fig. 1E–H) showed that there were no significant differences in intestinal microbial communities before and after the infection with *P. locustae* (*P* =0.1).

### Changes in the Composition of Intestinal Microbial Communities after *P. locustae* Infection

The results in Table 3 show that test insects in the infected and the control groups had similar compositions of intestinal microbial communities, and that bacterial communities had the highest abundance. Specifically, the Pseudomonadota had the highest abundance, followed by Bacillota, while Mucoromycota ranked third.

**Table 3.** Comparison of high-abundance intestinal microbial communities at the phylum and species levels before and after the infection of *C. italicus* with *P. locustae*

Sample	Abundance	Phylum	Percentage	Species	Percentage
**FI**	Top1	Pseudomonadota	67.41	*Enterobacter hormaechei*	11.15
	Top2	Bacillota	23.59	*Pediococcus acidilactici*	20.73
	Top3	Mucoromycota	2.14	*Rhizophagus irregularis*	45.88
**FC**	Top1	Pseudomonadota	43.32	*Enterobacter hormaechei*	7.69
	Top2	Bacillota	34.74	*Lactococcus garvieae*	43.10
	Top3	Mucoromycota	6.70	*Rhizophagus irregularis*	41.47
**MI**	Top1	Pseudomonadota	38.14	*Klebsiella pneumoniae*	13.52
	Top2	Bacillota	27.25	*Pediococcus acidilactici*	43.31
	Top3	Mucoromycota	9.30	*Rhizophagus irregularis*	43.37
**MC**	Top1	Pseudomonadota	35.75	*Solemya velum gill symbiont*	21.57
	Top2	Bacillota	29.81	*Virgibacillus salexigens*	52.57
	Top3	Mucoromycota	11.54	*Rhizophagus irregularis*	41.81

There were differences between males and females in the dominant species of intestinal microbes before and after infection. *Enterobacter hormaechei*, a species in the Pseudomonadota, had the highest relative abundance in female *C. italicus* both before and after infection, accounting for 7.69% and 11.15% of microbial communities, respectively. The dominant species of Bacillus was *Lactococcus garvieae* (43.10%) before infection, and *Pediococcus acidilactici* after infection (20.73%). After the infection of male *C. italicus* with *P. locustae*, *Klebsiella pneumoniae* (Pseudomonadota), a species strongly associated with insect diseases, became the dominant species, accounting for 13.52% of the diversity. *Rhizophagus irregularis* (Mucoromycota) was the dominant species before and after infection, accounting for more than 40% of the species diversity.

The test insects showed similar compositions of major intestinal microbial communities (bacteria, fungi, and viruses) before and after infection (Supplementary Fig. S1). In the bacterial communities of FI, FC, MI, and MC (Supplementary Fig. S1A), the Pseudomonadota and Bacillus occupied the absolute dominant positions, with relative abundances as high as 94.48%, 95.99%, 94.31%, and 92.68%, respectively. The compositions of intestinal fungal communities were similar (Supplementary Fig. S1B), and high-abundance *P. locustae* phyla appeared in the infected groups as well (FI, MI), accounting for 30.60% and 40.38%, respectively. Moreover, the proportions of microbial communities in the Mucoromycota, Basidiomycota, and Ascomycota in the infected groups (FI, MI) were lower than those in the control groups (FC, MC), respectively. By analyzing the composition of viral communities (Supplementary Fig. S1C), it was found that, in the infected groups (FI, MI) and the control groups (FC, MC), the Preplasmiviricota was always the dominant microbial phylum, accounting for 34.29%, 28.78%, 34.48%, and 27.29%, respectively; and that the relative abundances of the infected groups were slightly higher than those of the control groups. The relative abundances of some viral communities were gender-related. The Uroviricota had high relative abundances (30.96% and 25.00%) in females (FI, FC), while the Artverviricota had high relative abundances (27.36% and 28.35%) in males (MI, MC). The composition of archaeal communities was relatively simple (Supplementary Fig. S1D), and the Euryarchaeota appeared only in the infected groups (FI, MI), with relative abundances of 26.40% and 32.20%, respectively. The relative abundances of the Nitrososphaerota in the control groups (FC, MC) were higher than those in the infected groups, reaching 44.44% and 36.66%, respectively.

There were differences in the composition and relative abundance of intestinal microbes before and after infection (Supplementary Fig. S2). By analyzing the relative abundance of intestinal bacteria (Supplementary Fig. S2A), it was found that *Klebsiella aerogenes* (5.09%) and *Enterococcus faecalis* (3.57%), 2 typical pathogenic bacteria, appeared only in the infected groups (FI, MI). There were increases in the relative abundances of other opportunistic pathogenic bacteria, including *Klebsiella pneumoniae* and *Salmonella enterica*, while probiotics such as *Pe. acidilactici* increased significantly in the intestinal tract of infected insects (*P<*0.05). The relative abundances of probiotis such as *E. hormaechei* increased. By ­analyzing the relative abundance of fungal communities (Supplementary Fig. S2B), it was found that the relative abundances of *P. locustae* in the infected groups (FI and MI) were high, ie 21.43% and 26.58%, respectively. In viral communities (Supplementary Fig. S2C), a variety of Bacteriophage viruses, such as *Enterococcus phage*, appeared in the infected groups. In archaeal communities (Supplementary Fig. S2D), the dominant species was *Nitrososphaerota archaeon*, the relative abundances of which dropped significantly after infection (FI: 21.43%; MI: 18.18%) compared with the control groups (FC: 44.44%; MC: 26.27%).

UpSet analysis (threshold > 0.1) was performed to analyze changes in the species and populations of common and specific microbes in the intestinal microbial communities of *C. italicus* before and after *P. locustae* infection. There were a total of 1,314 microbes in FI and FC, including 274 and 62 specific ones, respectively. The MI and MC groups had a total of 1,042 microbes, including 324 and 80 specific ones, respectively. Analysis revealed that the populations of infection-specific taxa in male and female *C. italicus* after *P. locustae* infection were 4.42 and 4.05 times those in the control groups, respectively.

LEfSe analysis was performed to identify the indicator species (LDA > 4.0) of intestinal microbial communities before and after the infection of *C. italicus* with *P. locustae*. The results showed that there were differences in the composition of intestinal biomarker taxa between the infected and control groups. Five biomarker taxa were identified in FI, namely, *Klebsiella aerogenes*, *Lactococcus*, *Enterococcus faecalis*, *Enterobacter*, and *Enterococcus faecium*. Three biomarker taxa were identified in FC, namely, *Lactococcus garvieae*, *Virgibacillus salexigens*, and *Rhizophagus irregularis*. Five biomarker taxa were identified in MI, including *Pseudocitrobacter, Citrobacter koseri*, *Pseudocitrobacter faecalis*, *Enterobacteriaceae bacteria*, and *Salmonella enterica*. Finally, 2 biomarker taxa were identified in MC, namely, *Virgibacillus salexigens* and *Solemya velum gill symbiont*.

### Functional Differences of Intestinal Florae after *P. locustae* Infection

Taking into account the functional annotation information of microbial genomes at various KEGG levels, we explored the differences in the functions of the intestinal microbial communities of *C. italicus* before and after *P. locustae* infection (Table 4). The functions of intestinal microbes were annotated into 6 types of pathways, including Cellular Processes, Environmental Information Processing, Genetic Information Processing, Human Diseases, Metabolism, and Organismal Systems. Enriched gene annotation pathways were consistent before and after infection. More than 50% of the genes from intestinal microbes were annotated into Metabolism, while less than 3% of genes were annotated into Organismal Systems. The data showed that the number of genes annotated into Cellular Processes, Environmental Information Processing, Human Diseases, and Metabolism in the infected groups was higher than that in the control groups. The number of genes annotated into the Genetic Information Processing pathway in the infected groups was lower than that of the control groups.

**Table 4.** Functional annotations of intestinal microbes based on the KEGG database

KEGG metabolic pathway	Gene abundance			
	FI (%)	FC (%)	MI (%)	MC (%)
**Cellular processes**	4.24	3.34	4.44	3.66
**Environmental information processing**	5.79	5.53	6.10	5.49
**Genetic information processing**	4.83	6.91	5.46	5.67
**Human diseases**	3.50	3.36	8.08	4.02
**Metabolism**	57.24	51.29	55.95	51.93
**Organismal systems**	1.39	1.40	2.61	2.19
**None**	23.01	28.17	17.36	27.04

More than 70% of the total number of annotated microbial genes (excluding unannotated genes) were related to Metabolism, and the gene abundances of the infected groups (FI, MI) were significantly higher than those of the control groups (FC, MC) (*P<*0.05). At the level of secondary classification, a total of 15,239 metabolic pathways were enriched, where 12,782 of them were enriched in the infected groups, including carbohydrate metabolism, amino acid metabolism, cofactor and vitamin metabolism, energy metabolism, and nucleotide metabolism. The control groups were enriched with only 2,457 metabolic pathways, including terpenoids/polyketones metabolism, biosynthesis of other secondary metabolites, carbohydrate metabolism, energy metabolism, and glycan ­biosynthesis and metabolism. This suggests that *C. italicus* responded to the *P. locustae* infection by enhancing its microbial metabolic activities.

## Discussion

### Adaptive Changes in the Intestinal Microbial Community

The intestinal microbes of insects are affected by their feeding habits, interactions between microbes, environments (Smith et al. 2017, Zheng et al. 2021). Our research results showed that, notwithstanding the wide conservatism of the core intestinal microbiota of locusts is widely conserved, *P. locustae* infection induced adaptive changes in the composition of intestinal microbial communities in *C. italicus*, which validated hypothesis 1.

Before and after *P. locustae* infection, the core intestinal microbiota of *C. italicus*, such as Pseudomonadota, Bacillus, and Mucoromycota, remained stable. The core intestinal microbiota of *C. italicus* was consistent with those of *Oxya chinensis* (Thunberg, 1815) (Orthoptera: Acrididae), *Pararcyptera microptera meridionalis* (Ikonnikov, 1911) (Orthoptera: Acrididae), *Gastrimargus marmoratus* (Thunberg, 1815) (Orthoptera: Acrididae), and *Calliptamus abbreviatus* (Ikonnikov, 1913) (Orthoptera: Acrididae) (Ling et al. 2022), as well as 6 other species of locusts living in tall grass steppes (Muratore et al. 2020). This suggests that these phyla, as the core intestinal microbiota of locusts, are widely conserved and that the composition of intestinal microbes is relatively simple.

A *P. locustae* infection can lead to acidification of the intestinal tract of insects. Since an acidic environment is unsuitable for the growth of most bacteria, it will induce environmental selective stress (Tan et al. 2015). The results of this study confirmed that the evenness and diversity of the intestinal microbial communities in the host decreased after *P. locustae* infection, which is supported by the research of Tan et al. (2015) on *Locusta migratoria*. After the infection of male and female *C. italicus* with *P. locustae*, there were significant increases in the populations of infection-specific taxa, which indicates adaptive changes in intestinal microbes. The relative abundances of intestinal bacterial and fungal communities in male and female individuals presented different trends after *P. locustae* infection, which might be gender-related (Liu et al. 2025). Following infection with *P. locustae*, the relative abundance of bacteria and fungi at the phylum level showed similar trends in both female and male locusts. However, at the species level, the relative abundance exhibited different trends between the sexes. Given that significant differences exist in the  intestinal microbial composition between female and male insects (Liu et al. 2025), we speculate that this phenomenon is sex-related and exhibits sexual dimorphism.

Metagenomic functional annotation in this study revealed that *P. locustae* infection significantly reshaped the metabolic functions of the locust intestinal microbiota. In the control group, terpenoid and polyketide metabolic pathways were significantly enriched. Given that terpenoids play key roles in insect intraspecific and interspecific interactions (such as alarm signal transmission or defense compound synthesis), this finding suggests that, under steady-state conditions, the intestinal microbiota is primarily oriented toward the production of secondary metabolites. These compounds may help maintain host intestinal health by forming a barrier against invasion by foreign pathogens (Beran et al. 2019). In the infected group, however, microbial genes were highly enriched in core metabolic pathways, including carbohydrate, amino acid, energy, and nucleotide metabolism. This indicates that, in response to the physiological stress caused by *P. locustae* infection, the intestinal microbiota redirected metabolic resources from defensive secondary metabolism to fundamental metabolic and biosynthetic processes. As obligate intracellular eukaryotic parasites, microsporidia rely entirely on host cells for substrates and energy to complete their proliferation. Therefore, this metabolic shift may directly reflect the surge in energy demand during host–pathogen interactions (Zhang et al. 2023). The observed reduction in the relative enrichment of terpenoid and polyketide metabolism in the infected group does not necessarily imply a loss of their absolute functions; rather, it may result from a substantial increase in core metabolic pathways such as carbohydrate and amino acid metabolism, leading to a decreased functional proportion. The specific regulatory mechanisms underlying this shift warrant further investigation.

### The Interplay between Intestinal Pathogenic and Probiotic Bacteria Influences the Pathogenic Progression of *P. locustae* in Locusts

After the infection of *C. italicus* with *P. locustae*, the stability of its intestinal microbiota was destroyed, as manifested by a simultaneous increase in pathogenic bacteria and probiotics. The continuous interplay between pathogenic bacteria and probiotics in the intestinal tract delayed the pathogenesis of *P. locustae* in the host, which supported hypothesis 2.

From the perspective of competition for ecological niches, probiotics compete with pathogenic bacteria by increasing proliferation, thereby inhibiting the proliferation of pathogenic bacteria in the host (Douglas 2015). Results from this study confirmed this, where the metabolic activities of the intestinal microbes in *C. italicus* were significantly enhanced after *P. locustae* infection, which is presumed to be a coping strategy adopted by the host in response to *P. locustae* infection. In the infection stage, pathogenic bacteria establish a dominant ecological niche, and there were significant increases in multiple insect pathogenic bacteria, such as *Klebsiella aerogenes*, *Enterococcus faecalis*, and *Salmonella enterica*. Among them, *K. aerogenes* and *E. faecalis* were the biomarkers of the infected groups, as they were present exclusively in the infected samples. *E. faecalis* is resistant to the harsh conditions of the gastrointestinal tract, allowing it to gain a competitive advantage under host stress or dysbiosis (Du et al. 2021). *K. aerogenes* may be associated with intestinal inflammation resulting from infection. This bacterium is adept at utilizing metabolic byproducts of the inflammatory environment, such as nitrate, to promote its own proliferation (Xie et al. 2025). Increases in these opportunistic pathogenic bacteria eventually led to the death of the host. Studies have confirmed that *K. aerogenes* can accelerate the death of *Caenorhabditis elegans* by secreting toxins (Gowripriya et al. 2024). Subject to the pathological state of the insect host, *E. faecalis* can turn into an opportunistic ­pathogen (Mason et al. 2011).

Existing studies have also shown that probiotics protect the host and attenuate the pathogenicity of pathogenic bacteria by secreting antibacterial substances and activating host defense pathways (Borges et al. 2021, Gajger et al. 2023). The relative abundances of *Pe. acidilactici* in the males and females increased significantly by 3.5 and 1.5 times, respectively, after *P. locustae* infection. In the honeybee model, *Pe. acidilactici* could significantly prolong the life of insects stressed by *P. locustae* and pesticides (eg thiamethoxam and boscalid) (Peghaire et al. 2020). *Pe. acidilactici* can ferment to produce short-chain fatty acids such as lactic acid through the carbohydrate metabolism pathway, and directly participate in the tricarboxylic acid cycle, providing the host with a substrate for ATP synthesis (Harun et al. 2024). Similarly, *E. hormaechei* was identified exclusively in the gut of female *C. italicus*. This bacterium has been shown to inhibit the growth of pathogenic bacteria such as *Pseudomonas aeruginosa, Providencia shigelloides*, and *Providencia vermicola*, while simultaneously promoting the proliferation of beneficial bacteria, optimizing microbial community structure, and accelerating insect growth and development (Cirimotich et al. 2011). In *Anopheles gambiae* (Giles, 1902) (Diptera: Culicidae), *E. hormaechei* can disrupt the development of the parasite before *Plasmodium falciparum* invades the midgut epithelium of the host, protecting the host from infection (Boissière et al. 2012). Probiotics enhance insect growth, development, and disease resistance by regulating immune responses and energy metabolism (Jastaniah et al. 2024), thereby weakening the lethal effect of pathogenic bacteria and playing an important role in prolonging the life of the host.

In summary, the interaction between probiotics and pathogenic bacteria after *P. locustae* infection makes it difficult for *P. locustae* to quickly break through the defense system of the host, thus delaying its infection of the host. This discovery provides a new perspective for research on insect–pathogen interactions, laying the foundation for the development of microbiome-based strategies for the biological control of locusts. In the future, pathogenic strains or probiotics with clear effects on the intestinal microbiota should be identified through experiments.

## Supplementary Material

ieag027_Supplementary_Data
