# Supplementary material for: Host intestinal microbiota adaptive changes following Paranosema locustae infection and mechanism of chronic pathogenesis
Source: J Insect Sci. 2026 Mar 30;26(2):ieag027. doi: 10.1093/jisesa/ieag027 (PMC13035071; doi:10.1093/jisesa/ieag027)
Supplement: ieag027_Supplementary_Data [file ieag027_supplementary_data.zip › Legends of Supplementary Figure files.docx]

**Legends of Supplementary Figure files:**

**Figure S1:** Figure S1 Relative abundances of intestinal bacteria (A), fungi (B), viruses (C), and archaea (D) at the phylum level before and after the infection of *C. italicus* with *P. locustae.*

**Figure S2:** Figure S2 Relative abundances of intestinal bacteria (A), fungi (B), viruses (C), and archaea (D) at the species level before and after the infection of *C. italicus* with *P. locustae.*
