# Supplementary material for: Host intestinal microbiota adaptive changes following Paranosema locustae infection and mechanism of chronic pathogenesis
Source: J Insect Sci. 2026 Mar 30;26(2):ieag027. doi: 10.1093/jisesa/ieag027 (PMC13035071; doi:10.1093/jisesa/ieag027)
Supplement: ieag027_Supplementary_Data [file ieag027_supplementary_data.zip › Supplementary Table.docx]

**Supplementary Table**

**Table S1 Sample information for this study**

| Gender Group | Sample | | Weight（g） | | | |
| --- | --- | --- | --- | --- | --- | --- |
| Female | FI | Sample 1 | 0.7800 | 0.8100 | 0.8154 | 0.7163 |
|  |  | Sample 2 | 0.7375 | 0.7318 | 0.6990 | 0.7296 |
|  |  | Sample 3 | 0.7079 | 0.7471 | 0.7589 | 0.7523 |
|  | FC | Sample 1 | 0.7717 | 0.7359 | 0.7194 | 0.7685 |
|  |  | Sample 2 | 0.7252 | 0.6829 | 0.7958 | 0.8005 |
|  |  | Sample 3 | 0.7636 | 0.7188 | 0.7871 | 0.7592 |
| Male | MI | Sample 1 | 0.3082 | 0.3582 | 0.3410 | 0.3896 |
|  |  | Sample 2 | 0.3251 | 0.3715 | 0.3598 | 0.3514 |
|  |  | Sample 3 | 0.3984 | 0.3358 | 0.3802 | 0.3479 |
|  | MC | Sample 1 | 0.3456 | 0.3732 | 0.3418 | 0.3392 |
|  |  | Sample 2 | 0.3128 | 0.3491 | 0.3089 | 0.3419 |
|  |  | Sample 4 | 0.4034 | 0.3550 | 0.3405 | 0.3571 |

**TableS2 Raw and clean Reads from the sequencing data**

| Sample | Raw Date （bp） | Clean Date （bp） | Effective（%） | Q30 （%） | GC （%） |
| --- | --- | --- | --- | --- | --- |
| FI | 118989644 | 116324276 | 97.76 | 88.88 | 40.5 |
| FC | 114301727 | 107763668 | 94.28 | 91.25 | 40 |
| MI | 104953155 | 102329326 | 97.5 | 89 | 40.7 |
| MC | 116681876 | 113998193 | 97.7 | 89.50 | 40.8 |

**Table S3 Statistics of metagenomics sequencing data**

| Sample | Contig Num | ContigLen （bp） | N50 | MaxLen （bp） | AverageLen |
| --- | --- | --- | --- | --- | --- |
| FI | 1369105 | 732602887.7 | 530 | 158936.33 | 534.9 |
| FC | 1337039 | 713681511.3 | 531.33 | 54740 | 533.8 |
| MI | 1114009 | 586068141.7 | 517.67 | 236336 | 526 |
| MC | 1307224 | 689861865 | 523.33 | 96840.67 | 537.67 |
