# Supplementary figures and images for: Host intestinal microbiota adaptive changes following Paranosema locustae infection and mechanism of chronic pathogenesis
Source: J Insect Sci. 2026 Mar 30;26(2):ieag027. doi: 10.1093/jisesa/ieag027 (PMC13035071; doi:10.1093/jisesa/ieag027)

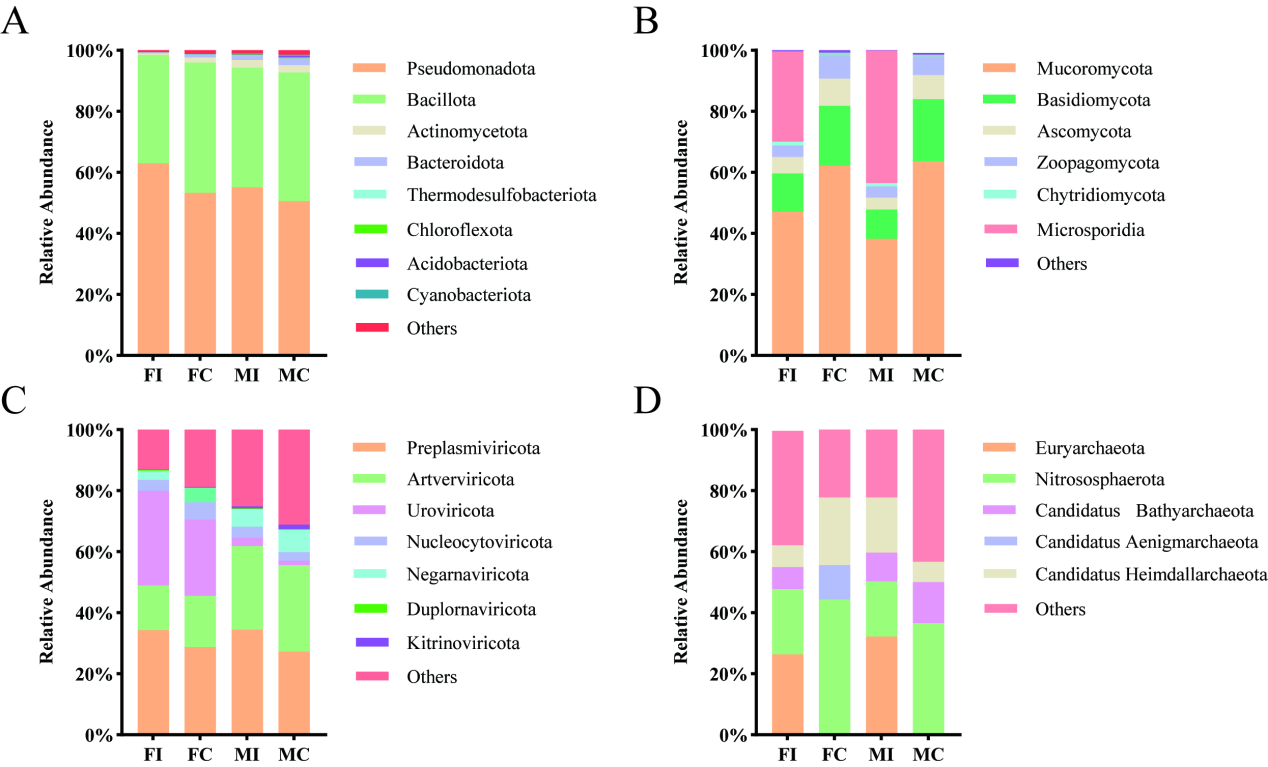

Supplement: ieag027_Supplementary_Data [file ieag027_supplementary_data.zip › Figure S1.docx]

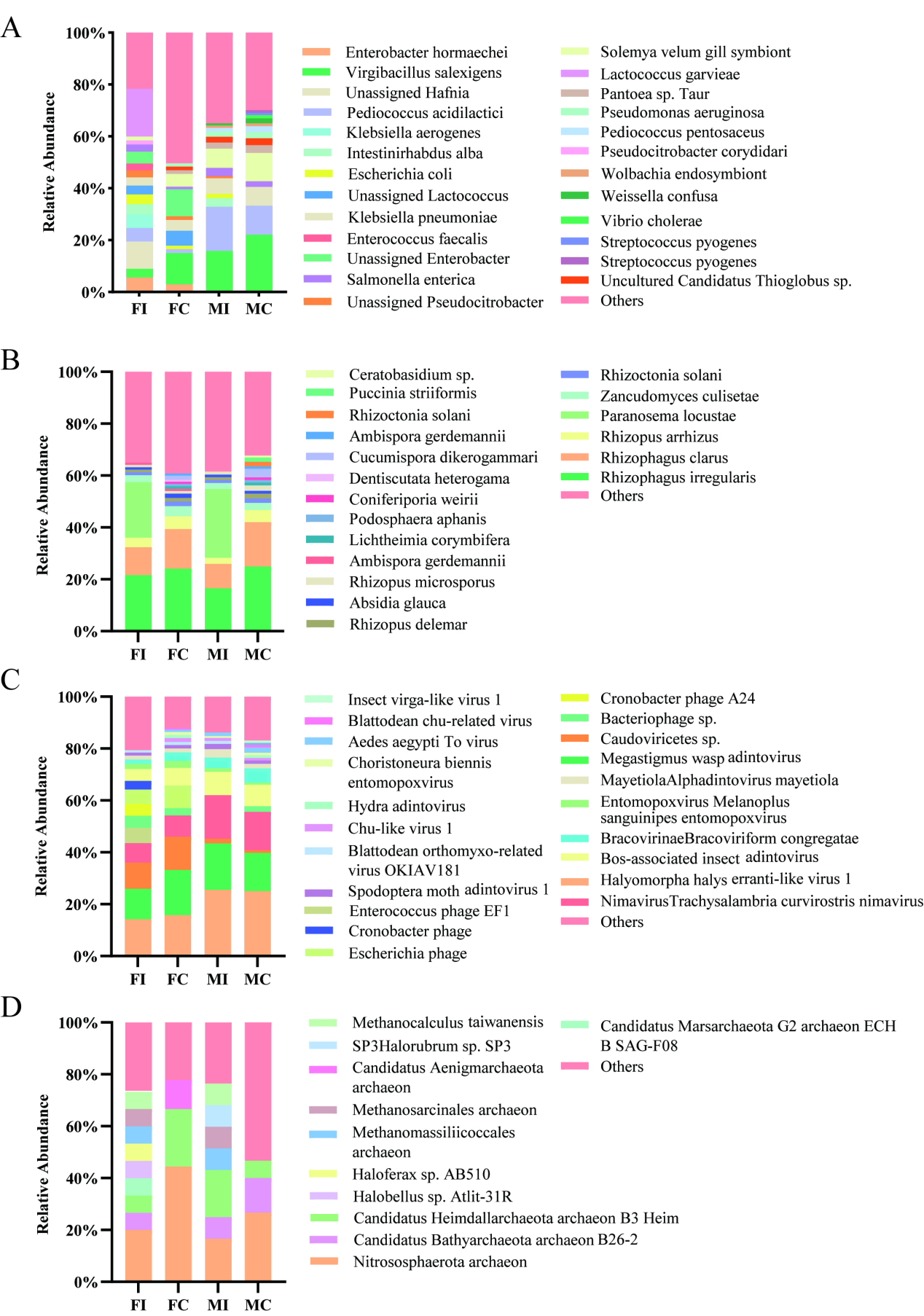

Supplement: ieag027_Supplementary_Data [file ieag027_supplementary_data.zip › Figure S2.docx]
